# Nutritional status in mentally disabled children and adolescents: A study from Western Turkey

**DOI:** 10.12669/pjms.292.3194

**Published:** 2013-04

**Authors:** Nalan Hakime Nogay

**Affiliations:** Nalan Hakime Nogay, Assistant Professor, Department of Nutrition and Dietetics, Kirklareli University College of Health, Kirklareli, Turkey.

**Keywords:** Children, Mental retardation, Nutrition

## Abstract

***Objective:*** To assess the nutritional status of mentally disabled children in Turkey on the basis of anthropometric indicators and dietary intake.

***Methodology:*** The sample of the study consisted of 77 mentally disabled children with ages between 10 and 18 years. The body mass index and body fat ratios of the children were calculated by measuring their body weight, height, and the skinfold thickness of their triceps and subscapular. Their three-day nutrition consumption was recorded in order to determine their nutrient intake.

***Results:*** When the body weights of the children were evaluated according to their ages, 14.3% of the children were found to be thin. The shortness rate among the participants was 28.6%. The shortness ratio was found to increase with age and girls had a higher inclination for shortness than boys. The ratios of those with ≤5^th^ percentile upper middle arm circumference were 32.7% in the 10-13 age group, and 36.0% in the 14-18 age group. The folic acid and calcium intakes of girls in the 10-13 age group, and the calcium intakes of boys in the 10-13 age group were under the suggested values. In the 14-18 age group the vit C and calcium intakes of girls and the calcium intakes of boys were under the recommended values.

***Conclusions:*** The prevalence of malnutrition is high among mentally disabled children. In order to raise their quality of life, mentally disabled children must be provided with sufficient nutritional support.

## INTRODUCTION

Mental retardation is a condition diagnosed before age 18 years that includes below average general intellectual function, and a lack of the skills necessary for daily living.^1^ Mental retardation is characterized by sub average intellectual functioning, existing concurrently with limitations in conceptual, social, and practical adaptive skills.^2^ Mental retardation is a clinically and socially important condition.^3^ Under nutrition may influence brain development by directly affecting brain processes or indirectly by affecting children’s experiences and behavior.^4^ Disabled children are known to be at high risk for developing malnutrition, which may partly explain the growth retardation often encountered in such children.^5^ The most common problems associated with malnutrition in disabled children, are inadequate nutrient intake either due to feeding problems or poor feeding knowledge among care providers.^6^

Mental retardation affects 3% of the total pediatric population.^7^ According to the results of the Disabilities in Turkey study, the ratio of the disabled population in the normal population is 12.29%, and 15.5% of the disabled population is mentally disabled.^8^

The aim of the present study was to assess the nutritional status of mentally disabled children in Turkey on the basis of anthropometric indicators and dietary intake.

## METHODOLOGY

The study was conducted with 77 children with mental retardation between the ages of 10-18 who attended a specialized education and rehabilitation center as well as an applicational education and vocational school.

***Anthropometric measurement: ***Body weight and height: Body weight was measured with a scale sensitive to ± 0.1 kg with thin clothing and no shoes, whereas height was measured with the feet adjacent and the head on a Frankfort plane.

***Body mass index:*** Body mass index was calculated through the Weight(kg)/height^2^(m^2^) formula.

***Triceps skinfold thickness:*** The arm was released after marking the midpoint between the shoulder and elbow protrusions when the left arm was bended 90^o^ from the elbow, the layer was held with the left index finger by going upward from the elbow parallel to the epicondillia, and the measurement was made with the right hand using a caliper.

***Subscapular skinfold thickness:*** The inferior corner of the left scapula bone was marked, the layer was held with the left hand in a 45^o^ angle to the spine, and the measurement was made using a caliper. The anthropometric measurements of the children were evaluated according to age and gender.^9^

***Body Fat Ratio:*** The body fat ratios of the individuals were calculated using the Slaughter Equations.^10^ The amounts of macro and micro nutrients received through the diet have been assessed by calculating the data obtained from the three-day food consumption record using BeBis (Food Information System) Food Consumption Analysis Program.^11^

***Ethics Committee Approval:*** The study was approved by the Governor’s office of Kirklareli.

***Statistical Analysis*** ***:*** Data obtained were assessed with SPSS 15.0 statistics software package.^12^ T-test was used to test the significance of the difference between two groups Significance level in these tests was determined to be < 0.05.

## RESULTS

Among the kids participating in the study, 27 had mild mental disability, while 30 had medium and 20 had severe mental disability. The height and waist circumference averages of the boys in the 10-13 age group was higher than those of the girls. The averages of the triceps skinfold thickness (TSF), subscapular skinfold thickness (SSF), and body fat ratio of the girls in this group, however, were higher than boys although they showed no statistical difference. The body weight and height of boys in the 14-18 age group were higher than girls, while their upper mid arm circumferences TSF, waist circumferences and body fat ratios were lower, although no statistical difference was found (Table-I).

As seen in Table-II, the prevalence of thin individuals was 14.3%. Boys were (17.4%) thinner with regard to girls (9.4%), whereas the younger age group was thinner than the older age group. The shortness ratio was found to increase with age and girls had a higher inclination for shortness than boys.

**Table-I:** The averages of the anthropometric measurements according to age and gender

	*10 – 13 Years*			*14 – 18 Years*		
	*Male* *n= 31*	*Female* *n=21*	^†^ * p*	*Male* *n=15*	*Female* *n=10*	^†^ * p*
Weight (kg)	33.89±8.51	33.72 ±14.34	0.051	63.38±14.02	61.43±14.32	0.804
Height (cm)	137.35± 10.14	136.37±13.90	0.117	160.71±9.65	156.20±9.19	0.521
Upper mid arm circumferences (cm)	20.48 ±3.70	20.26 ±3.70	0.913	23.27±5.75	26.67±5.36	0.649
Triceps skinfold thickness(mm)	12.74± 5.83	13.69 ±5.65	0.958	14.79±5.73	18.12±5.92	0.657
Subscapular skinfold thickness(mm)	8.87 ±7.37	10.97±6.62	0.584	14.61±9.35	15.75±11.14	0.921
Body mass index (kg/m^2^)	17.70± 3.24	17.54±5.18	0.056	24.72±4.58	24.29±5.85	0.291
Body fat ratio (%)	20.38± 9.50	22.20±7.73	0.841	24.82±11.63	27.48±8.91	0.306

^† ^ t-test          *p < 0.05

**Table-II:** The evaluation of nutritional statuses according to anthropometric measurements

	*10 – 13 Years*	*14 – 18 Years*	*Male*	*Female*	*Total (77)*
	*52*	*25*	*46*	*31*	*n (%)*
	*n (%)*	*n(%)*	*n(%)*	*n(%)*	
*Weight for Age*					
Z Skor					
Under weight < - 2SD	8(15.4)	3(12.0)	8(17.4)	3(9.4)	11(14.3)
Overweight > + 2SD	1(1.9)	2(8.0)	2(4.3)	1(3.1)	3(3.9)
*Height for Age*					
Z Skor					
Stunted < - 2SD	13(25.0)	9(36.0)	10(21.7)	12(37.5)	22(28.6)
Tall > + 2SD	1(1.9)	-	-	1(3.1)	1(1.3)
*MUAC for Age*					
Persentil					
≤ 5^th^	17(32.7)	9(36.0)	17(36.9)	9(28.1)	26(33.8)
≥ 95^th^	2(3.8)	1(4.0)	2(4.3)	1(3.1)	3(3.9)
*TSF* * for age*					
Persentil					
≤ 5^th^	2(3.8)	-	1(2.2)	1(3.1)	2(2.6)
≥ 95^th^	2(3.8)	1(4.0)	3(6.5)	-	3(3.9)
*BMI for age*					
Persentil					
≤ 5^th^	6(11.5)	1(4.0)	2(4.3)	5(15.6)	7(9.1)
≥ 95^th^	5(9.6)	7(28.0)	8(17.4)	4(12.5)	12(15.6)

**Table-III:** The evaluation of the daily nutritional element intakes of the children and their adequacy

	*10 – 13 Years*				*14 – 18 Years*			
	*Male*		*Female*		*Male*		*Female*	
	*Mean* *(SD)*	*% * *Adequacy* *(SD)*	*Mean* *(SD)*	*% * *Adequacy* *(SD)*	*Mean* *(SD)*	*% * *Adequacy* *(SD)*	*Mean* *(SD)*	*% * *Adequacy* *(SD)*
Energy(kcal)	1540.3 (302.6)	62.9 (12.3)	1810.0 (296.2)	82.2 (13.4)	1985.4 (752.2)	69.4 (26.3)	1946.2 (409.6)	84.3 (15.5)
Protein(g)	60.80 (21.2)	123.87 (43.3)	67.1 (23.1)	159.8 (55.0)	79.4 (29.7)	126.0 (47.2)	74.5 (14.9)	135.5 (27.1)
Calcium (mg)	570.40* (335.3)	43.8 (25.7)	421.6 (70.4)	32.4 (5.4)	587.9 (236.3)	45.6 (17.5)	502.5 (190.5)	38.6 (14.6)
Zinc (mg)	8.4 (2.0)	76.6 (18.9)	8.8 (3.2)	88.2 (32.3)	10.8 (4.1)	98.9 (37.6)	10.0 (2.7)	100.8 (27.1)
Iron (mg)	10.26 (3.4)	102.5 (34.8)	10.0 (2.4)	100.2 (24.9)	14.3 (6.8)	143.9 (68.1)	14.1 (2.2)	78.5 (12.4)
Vitamin A(mcg)	603.6* (480.9)	100.6 (80.1)	548.3 (251.1)	63.7 (28.9)	477.0 (164.3)	79.5 (27.3)	625.8 (226.5)	89.3 (32.3)
Vitamin C(mg)	54.1 (42. 9)	72.1 (57.2)	68.4 (40.4)	91.2 (53.9)	65.4 (54.6)	77.8 (73.7)	36.5 (26.4)	48.7 (35.2)
Vitamin B_1_(mg)	0.73 (0.17)	81.9 (19.9)	0.6 (0.1)	68.8 (15.5)	0.9 (0.5)	80.8 (42.7)	0.8 (0.1)	86.2 (18.4)
Vitamin B_2_(mg)	0.9* (0.5)	110.1 (59.2)	1.0 (0.2)	113.5 (27.1)	1.1 (0.4)	86.7 (36.4)	1.0 (0.2)	106.4 (27.8)
Folic acid (mcg)	212.4 (101.8)	70.7 (33.9)	203.0 (47.7)	50.7 (11.9)	338.2 (222.9)	84.5 (55.7)	325.3 (81.8)	81.3 (20.4)
Niasin (mg)	11.2 (9.19)	93.5 (76.60)	11.9 (7.7)	99.7 (64.9)	12.1 (10.7)	75.8 (67.5)	10.6 (4.7)	76.1 (33.6)

^† ^ t-test          *p < 0.05

The ratios of those with ≤5^th^ percentile mid upper arm circumferences (MUAC) were 32.7% in the 10-13 age group, and 36.0% in the 14-18 age group. The ratio of those with a ≤5^th^ percentile MUAC was higher among boys compared to girls.

According to age, the ratio of those with TSF ≤ 5^th^ percentile was 2.6%, while the ratio of those with TSF ≥95^th^ percentile was 3.9%. The prevalence of obesity was higher in the higher age group than the younger, and in boys with regard to girls (Table-II). The body fat ratios of boys were lower than girls in both age groups. The ratio of obesity according to BMI was 15.6%. Obesity increased with age and were higher among more males than females (Table-II).

The energy, protein, vit C, zinc, folic acid and niacin intakes of girls were higher than that of boys in the 10-13 age group. In the 14-18 age group, all the nutritional element intakes of boys were higher than girls, except for vit C. In the 10-13 age group, the folic acid and calcium intakes of girls and the calcium intakes of boys were below the recommended values. In the 14-18 age group, the vit C and calcium intakes of girls and the calcium intakes of boys were below the recommended values (Table-III).

## DISCUSSION

According to the results of this study, when the body weights of the kids were evaluated according to age, 14.3% of the kids were thin. Boys were (17.4%) thinner with regard to girls (9.4%), whereas the younger age group (15.4%) was thinner than the older age group (12.0%). In a study conducted in Egypt with 639 mentally disabled children, 14.1% of the children were similarly found to be thin, and boys were found to be thinner than girls.^5^

The prevalence of obesity in this study, based on weight according to age, is 3.8%. In a study conducted with 279 mentally retarded children and adolescents, the prevalence of obesity was found to be 18.0%.^13^

Stunting is a nutrition problem that affects mainly developing countries.^14^ Poor linear growth, or stunting, in young children is the result of multiple circumstances and determinants, including antenatal, intra-uterine and postnatal malnutrition.^15^ Stunting in early life is associated with adverse functional consequences, including poor cognition and educational performance.^14^ According to our study, 18.6% of the children were stunted. The ratio of stunting increased with age, and girls (37.5%) were more inclined to become stunted than boys (21.7%). A study made on the subject with children without disabilities has reported the stunting ratio as 36.6%.^16^ In a study where the nutritional statuses of children with disabilities were evaluated, the ratio of stunting was reported to be 33.5% among all children, and the ratio of stunting was reported to increase with age, where girls were more inclined to become stunted than boys.^5^

Obesity in persons with developmental disability can contribute to the development of chronic diseases such as diabetes, hypertension and heart disease.^17^ In our study the ratio of obesity according to BMI was 15.6%, obesity increased with age and was more prevalent among males than females. Other studies have similarly found the ratio of obesity as 14%, and reported an increase in higher ages.^18^ In another study where the prevalence of obesity was evaluated based on body mass index in mentally disabled children, the prevalence of obesity was found to be higher in girls with regard to boys, which contradicts our study.^19^

Skin-fold thickness is a practical and valuable method used in evaluating both the nutritional status and the body adiposity.^20^ According to our study, the mean TSF and SSF values of girls were higher than boys in both age groups. In a study conducted with 6917 Turkish children and adolescents with no disabilities, the mean triceps and SSF values of girls were found to be higher than boys in all age groups, which support our findings.^21^

The major determinants of MUAC are muscle and sub-cutaneous fat, both important determinants of survival in malnutrition and starvation.^22^ According to our study, there was a decrease in the muscle and fat storages of 38.8% of the children, and the decrease was more pronounced in the higher age group with regard to the younger age group, and in girls with regard to boys. In a study conducted with mentally disabled children, similar results were found.^5^

Studies have shown strong relationships between body fat ratio and obesity as well as obesity related diseases in children and adolescents.^23^ Body fat levels ranging from 20%–25% in boys and 30%–35% in girls have been shown to be associated with health risk.^24^ In a study conducted on children without disabilities, body fat ratio averages were found to be 17.6% in boys and 23.9% in girls. Similarly, in our study, the body fat ratio of boys were found to be lower than girls in both age groups.

In our study, in the 10-13 age group, the folic acid and calcium intakes of girls and the calcium intakes of boys were below the recommended values. In the 14-18 age group, the vitamin C and calcium intakes of girls and the calcium intakes of boys were below the recommended values. In a study conducted on 17 children with refractory epilepsy, the calcium, zinc, iron, vitamin B_1_, vitamin B_2_ and niacin intakes of the children were reported to be below the recommended values.^25^ In a study evaluating the nutritional statuses of 50 children with mental retardation, the protein and vitamin B_1_ consumption of the children were found to be higher than the recommended values in all age groups.^1^ The protein consumption of the children were found to be higher than the recommended values in all age groups in our study as well.

## CONCLUSION

Malnutrition as revealed by anthropometric variables and micronutrient deficiency occurs with a high prevalence among mentally disabled children and almost increased with age. The nutritional statuses of mentally disabled children should be monitored closely, and sufficient nutritional support should be provided in order to ascertain a normal body weight, linear growth, and a higher quality of life.

## Authors’ Contributions

**NHN:** Completed the study design, manuscript writing, data collection, statistical analysis and editing manuscript.
